# Enhanced near-surface ozone under heatwave conditions in a Mediterranean island

**DOI:** 10.1038/s41598-018-27590-z

**Published:** 2018-06-15

**Authors:** Andri Pyrgou, Panos Hadjinicolaou, Mat Santamouris

**Affiliations:** 1 Energy, Environment and Water Research Center, The Cyprus Institute, P.O. Box 27456, Nicosia, 1645 Cyprus; 2 The Anita Lawrence Chair in High Performance Architecture, School of Built Environment, University of New South Wales, Sydney, 2052 Australia

## Abstract

Near-surface ozone is enhanced under particular chemical reactions and physical processes. This study showed the seasonal variation of near-surface ozone in Nicosia, Cyprus and focused in summers when the highest ozone levels were noted using a seven year hourly dataset from 2007 to 2014. The originality of this study is that it examines how ozone levels changed under heatwave conditions (defined as 4 consecutive days with daily maximum temperature over 39 °C) with emphasis on specific air quality and meteorological parameters with respect to non-heatwave summer conditions. The influencing parameters had a medium-strong positive correlation of ozone with temperature, UVA and UVB at daytime which increased by about 35% under heatwave conditions. The analysis of the wind pattern showed a small decrease of wind speed during heatwaves leading to stagnant weather conditions, but also revealed a steady diurnal cycle of wind speed reaching a peak at noon, when the highest ozone levels were noted. The negative correlation of NOx budget with ozone was further increased under heatwave conditions leading to steeper lows of ozone in the morning. In summary, this research encourages further analysis into the persistent weather conditions prevalent during HWs stimulating ozone formation for higher temperatures.

## Introduction

Air pollution has gained a growing interest the past decades due to its negative effects on human health; specifically cardiovascular and respiratory related diseases^1–6^. EU has set a guideline of 120 μg/m^3^ for a maximum daily 8-hour average exposure to near-surface ozone^7^. High ozone concentrations exceeding the EU standards have been frequently observed in urbanized cities over the globe^8–10^.

Mechanisms for near-surface ozone formation and depletion are complex and include dry and wet deposition, vertical and horizontal advection^11,12^ and chemical reactions of atmospheric volatile organic carbons(VOCs), nitrogen oxides (NOx), oxygen (O_2_), carbon monoxide (CO) in the presence of NOx and sunlight of wavelengths less than 240 nm^3,13,14^. Solar radiation (UVA and UVB) and high temperatures play a catalytic role in chemical reactions of ozone formation, having a strong positive correlation with ozone concentration values^9,15–18^. Previous studies have shown that ozone production accelerates at high temperatures (without changing VOC or NOx conditions)^19,20^, which may be attributed not only to the temperature dependence of chemical reactions, but also to the weak winds which accompany high temperatures and heatwaves and cause the atmosphere to stagnate and built up ozone levels^21^. Ozone does not only depend on the quantities of the precursors, but also on the ability of the atmosphere to form or deplete ozone and specifically under favourable meteorological conditions such as high temperature, intense solar radiations, long sunshine hours and low wind speed/direction, all characteristics of heatwaves.

Heatwaves (HWs) are defined by prolonged high temperatures and are generally characterized by the downward air movement and increased pressure favouring stagnant conditions, anticyclonic circulation and lower horizontal advection^22^. Ozone concentration levels depend greatly on atmospheric horizontal transport, especially near the sea and vertical transport associated with stratospheric-tropospheric exchanges showing significant seasonal and zonal variations^23–25^. Therefore the increase of frequency of heatwaves during the past decades urges the need for identification of the influencing meteorological and anthropogenic factors in ozone spikes in each region. This is becoming more important to regions like the eastern Mediterranean and Middle East where temperatures are elevated during summer and heatwaves are projected to become more severe in the future under anthropogenic climate change^26–28^.

Pu *et al*.^16^ studied the correlation more closely between ozone and temperature in China observing an increase rate of ozone concentration of about 4.5 ppb/K within a temperature range of 28–38 °C and a decreasing trend of about −1.5 ppb/K for temperatures over 38 °C^16^. Ooka *et al*.^12^ argued that higher temperatures and calm weather conditions (low wind speed) promoted ozone formation and limited ozone dispersion in urban areas whereas in rural areas the downward vertical advection enhanced ozone levels^12^.

Recent studies in the Mediterranean showed steady ozone levels over the past 20 years, and sudden spikes of ozone concentration levels attributed to decreased NOx concentration levels and more frequent biomass fires^9,10^. Kleanthous *et al*.^9^ suggested the limited role of local photochemistry and the strong role of long-range transport in ozone levels from the concurrent comparison of the annual cycles of solar radiation flux, nitrogen oxides and carbon monoxide over a rural location in Cyprus^9^. Specifically, a positive correlation of wind speed and temperature and a negative correlation of RH were indicative of the changes of the boundary layer height induced by the rise of hotter air at noon and the mixing with dryer air masses^9^.

The outlined research background highlighted the research behind air quality and UV radiation for photolysis. Motivation for studying the near-surface ozone concentrations arises directly from the correlation made by other studies on their hazardous effect on human health and dependence on temperature^2–4,9,17^. The purpose of the present study is to (i) present the seasonal and diurnal concentrations of near-surface ozone in Nicosia and (ii) observe how air-quality and meteorological parameters affect ozone levels in order to provide pioneering insight into variations in ozone measurements between heatwave and non-heatwave periods.

## Results

### Ozone under mean conditions

**Figure 1:**
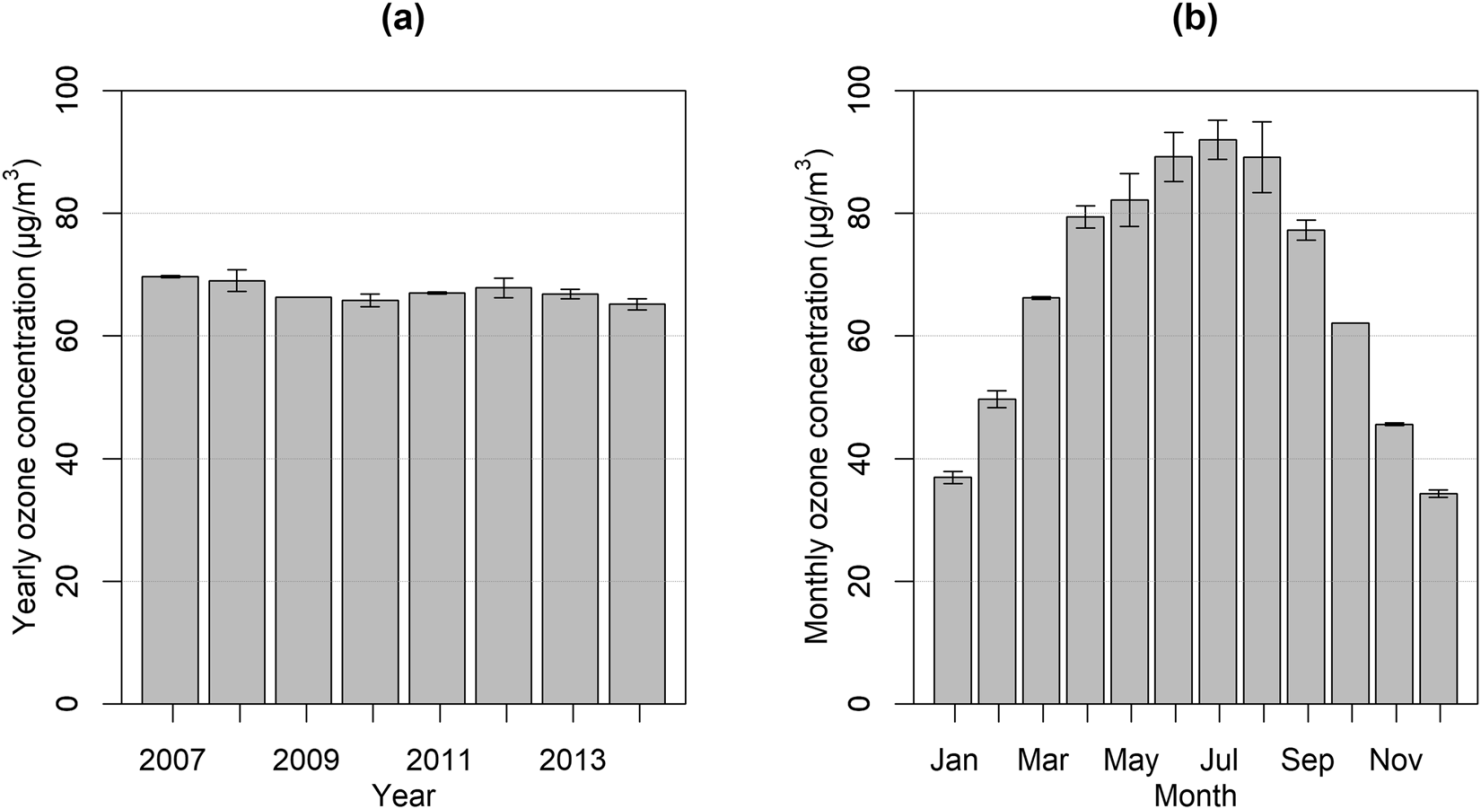
(**a**) Yearly and (**b**) monthly ozone concentration levels for years 2007–2014 with added error bars.

Figure 1a shows the annual mean time-series of the ozone concentration level noting a small decreasing trend over the years 2007–2014 for Nicosia, with a mean yearly value of 67.16 μg/m^3^ ± 3.5%. Figure 1b shows the annual cycle of the monthly mean ozone concentrations noting a maximum of 92.00 μg/m^3^ in July and a minimum of 34.24 μg/m^3^ in December. For the period 2007–2014, 79 days exceeded the EU limit, with the great majority (84.8%) of these exceedances occurring in the summer months, 12.7% (10 days) in spring and 2.5% in autumn.

**Figure 2:**
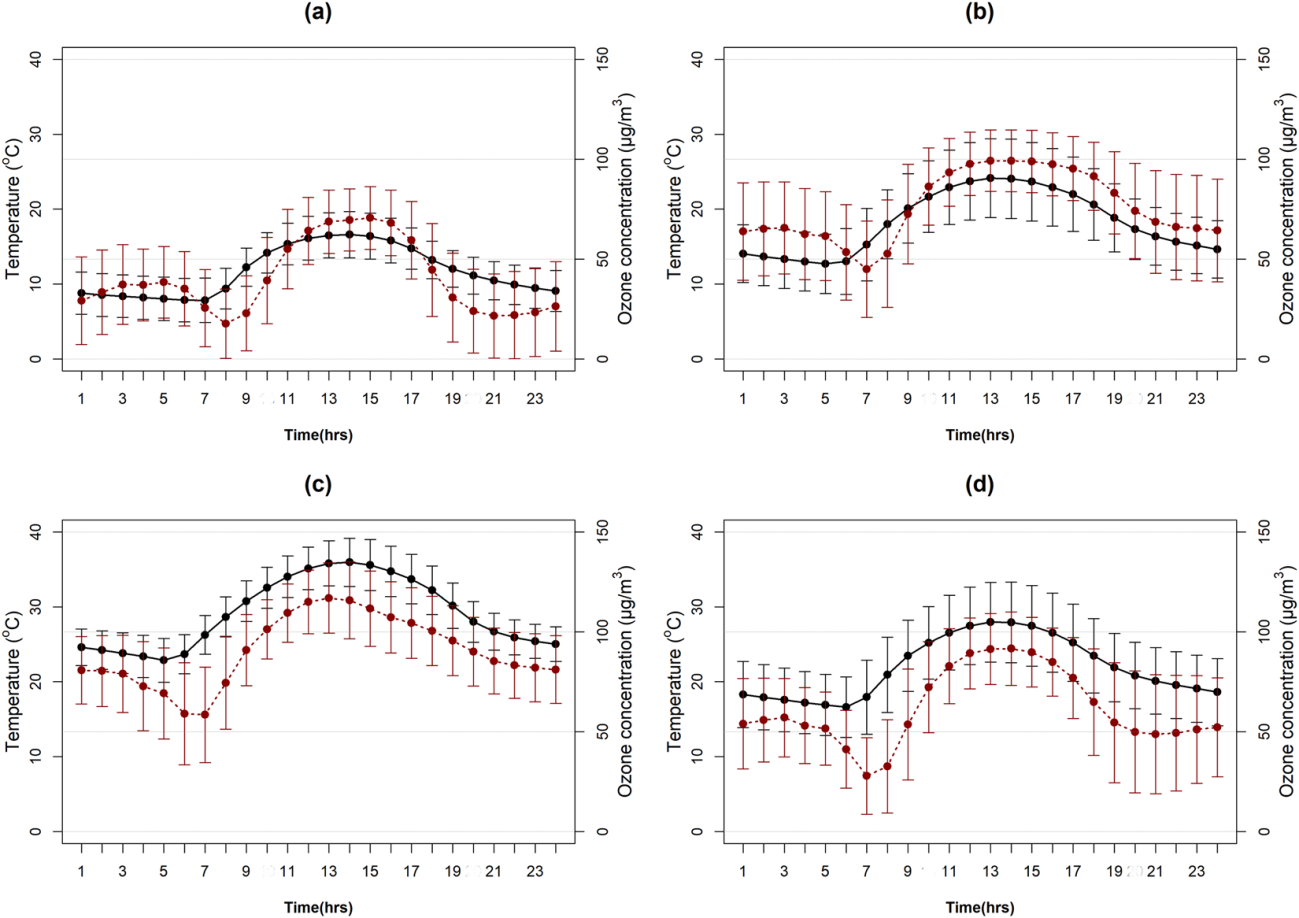
Diurnal (2007–2014 average) temperature (black solid line) and ozone concentration (dark red dotted line) for (**a**) winter (DJF), (**b**) spring (MAM), (**c**) summer (JJA) and (**d**) autumn (SON) with error bars of one standard deviation.

Figure 2 shows the climatology (2007–2014 average) of diurnal variation of ozone and nearby temperature for the four seasons. In Nicosia, the ozone concentration levels were higher for spring and summer. It is evident that the lowest temperature and ozone values occurred for all seasons in the morning (at 7:00 am and 8:00 am respectively) and the highest around noon (1:00 pm local time).

### Ozone under HW conditions

Heatwaves (HWs) were defined as the events of 4 consecutive days with daily maximum temperature over 39 °C. According to the proposed definition seven heatwave events were found for the period 2007–2014 in Nicosia, listed in Table 1.

**Table 1:** Recorded heatwave events from 2007–2014.

Event no	Start date	End date	Duration (days)
1	23.07.2007	31.07.2007	9
2	21.07.2008	24.07.2008	4
3	2.08.2008	6.08.2008	5
4	15.08.2010	21.08.2010	7
5	13.07.2012	20.07.2012	8
6	11.08.2014	15.08.2014	5
7	22.08.2014	26.08.2014	5

**Figure 3:**
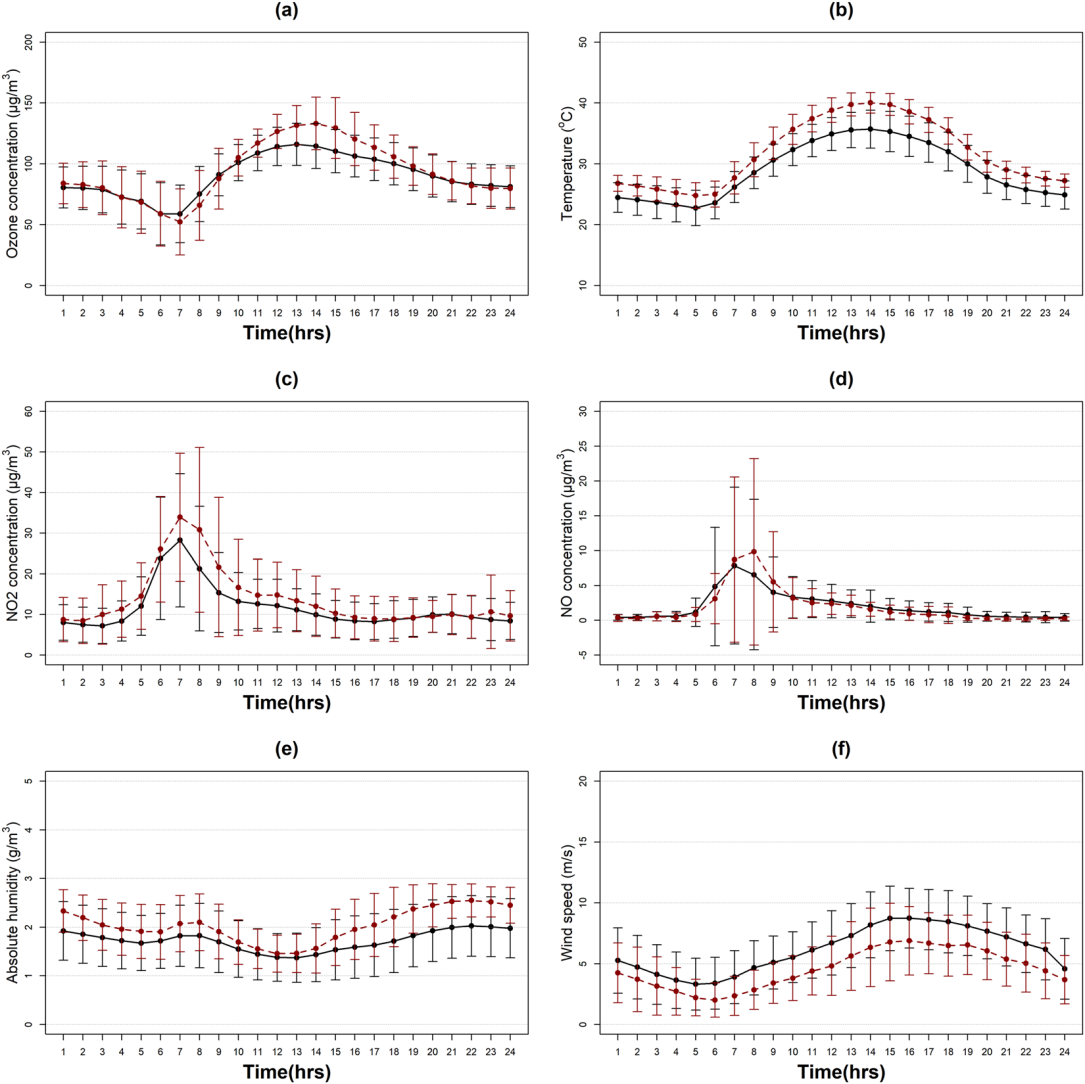
Diurnal variation under HW conditions (red dotted line) and the rest of the summer (black solid line) for (**a**) ozone, (**b**) temperature, (**c**) NO_2_, (**d**)NO, (**e**) absolute humidity and (**f**) wind speed with error bars of one standard deviation.

The diurnal variation of ozone measurements and other parameters was averaged (Fig. 3a–f) for the above listed heatwaves (HW period) and the rest of the summer periods (non-heatwave (NHW) periods). The strong relationship between near-surface ozone and temperature was noticeable, which increased under HW conditions exceeding the EU recommended levels from 11:00 am until 4:00 pm. Early morning ozone measurements were lower under HW conditions compared to NHW conditions. NOx precursors were higher under HW conditions, especially from 6:00 am until 10:00 am. Temperature showed a typical diurnal cycle with a minimum at night and a maximum at noon. The peak of absolute humidity was at night-time, whereas its lowest value was observed at noon. Small spike of absolute humidity levels at 7:00 am coincided with higher NOx budget and lower ozone measurements. The investigated HWs were characterized by lower wind speed, higher absolute humidity and temperature throughout the day, and increased NOx concentrations at 7–8 am coinciding with decreased ozone levels.

**Figure 4:**
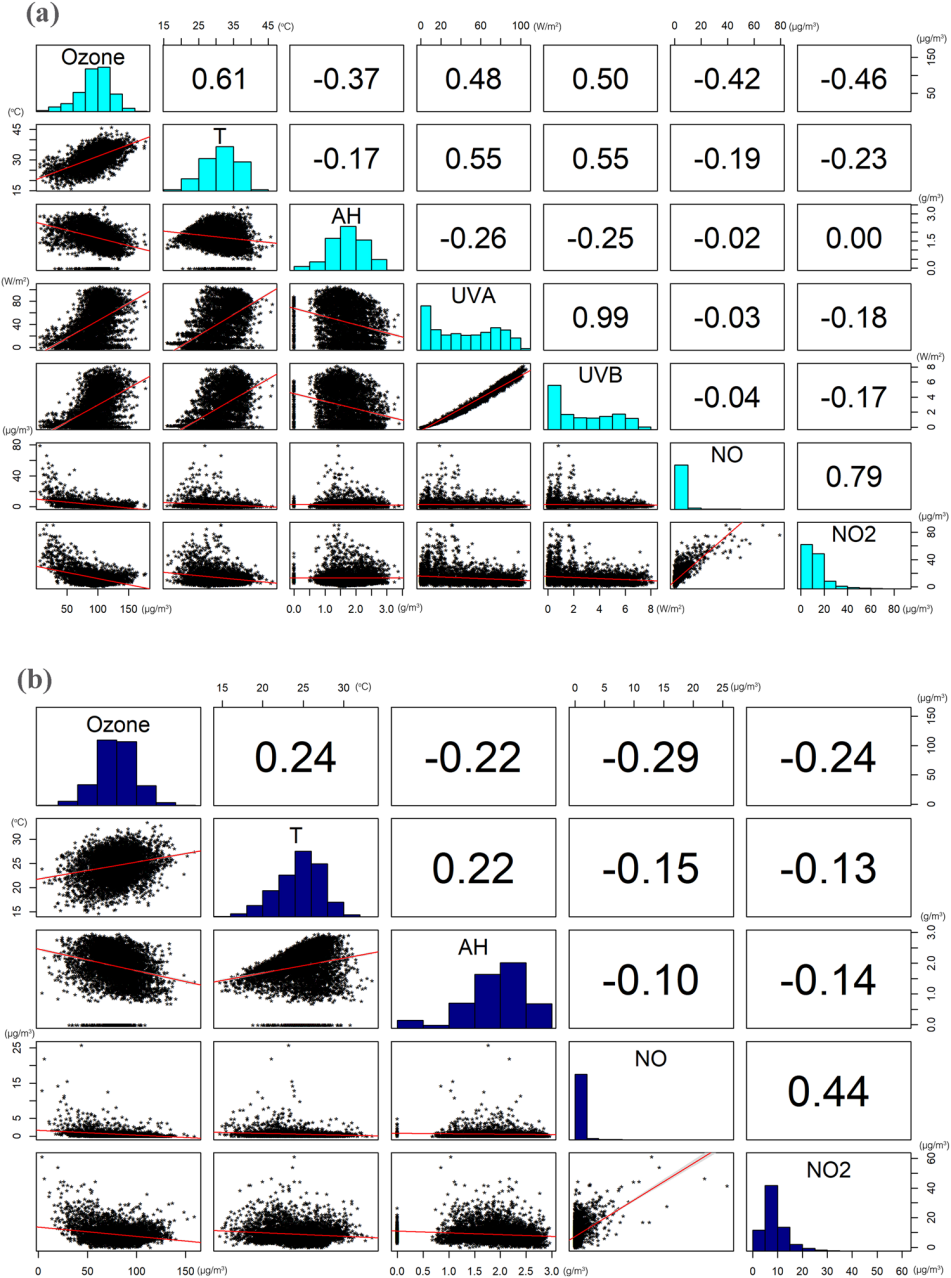
Correlation coefficients of available parameters for (**a**) daytime and (**b**) night-time for NHW periods.

Near-surface ozone is subject to local chemical reactions (CHEM) and physical processes. According to Fig. 3a, ozone concentration levels were increased during daytime under HW conditions. To investigate the variability of ozone concentration levels under HW and NHW periods for daytime (6:00 am-8:00 pm) and night-time (8:00 pm-6:00 am) we performed a correlation analysis of all the parameters; temperature (T), absolute humidity (AH), ultraviolet radiation (UVA & UVB) and its precursors NO and NO_2_, presented in Fig. 4 (for NHW conditions) and 5 (for HW conditions). According to this correlation analysis temperature, UVA, UVB had a positive correlation with ozone and were therefore considered as “enhancement factors”, whereas absolute humidity and NOx had a negative correlation and were considered as “reduction factors”.

**Figure 5:**
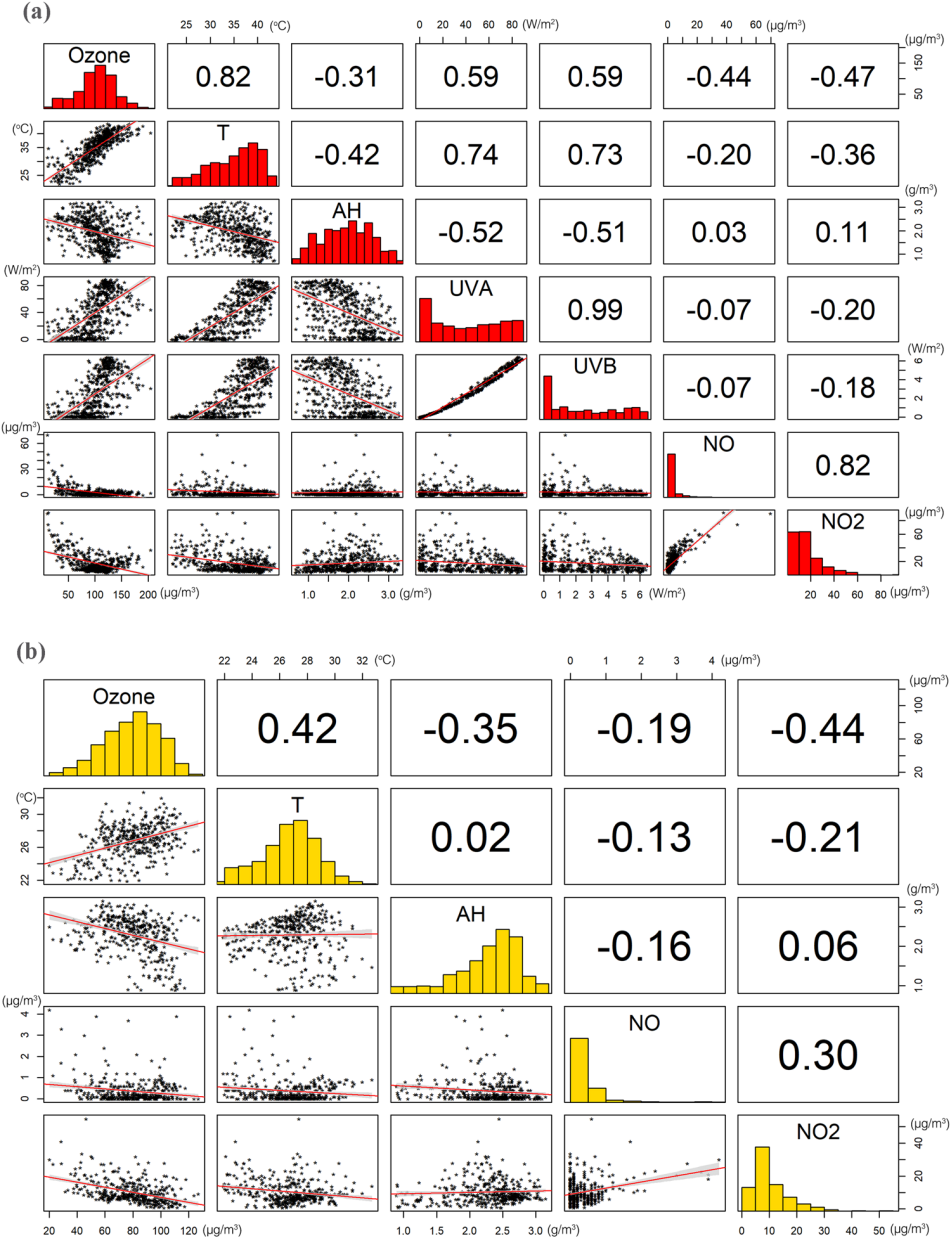
Correlation coefficients of available parameters for (**a**) daytime and (**b**) night-time for HW periods.

The results, revealed a noteworthy positive correlation of ozone with temperature (r = 0.61), and somewhat smaller with UVA (r = 0.48) and UVB (r = 0.50) at daytime during NHW days (Fig. 4a) which increased by about 1/3 under HW conditions (Fig. 5a). Positive but smaller correlation of temperature and ozone (r = 0.24) was also found at night-time (Fig. 4b), again enhanced (r = 0.42) during HW conditions. A negative correlation (r = −0.37) of absolute humidity with ozone was also found which did not vary under HW conditions.

The NO and NO_2_ correlation analysis noted a medium negative correlation with ozone (r = −0.42 and r = −0.46 respectively) which did not vary greatly during HWs. At night-time this correlation was considerably decreased.

Study of the vertical advection (VADV) effect on ozone measurements required the analysis of synoptic weather maps throughout the summer, showing a weak pressure gradient over Cyprus for both NHW and HW periods. The existent sparse isobaric high pressure lines led to an outward movement of the light air from the centre and consequently air from higher heights descended creating stationary high temperature conditions.

**Figure 6:**
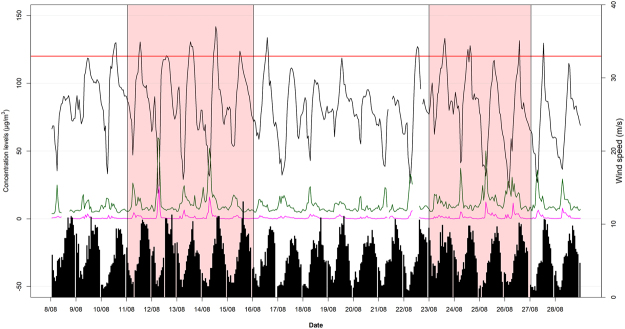
Diurnal profile of ozone (black line), NO (purple line) and NO_2_ (green line) concentration levels and wind speed (black bars) for period 8.08.2014–28.08.2014. The two HWs (11.08–16.08 & 23.08–27.08) are shaded in the red background.

Furthermore, the horizontal advection (HADV) was studied for the period 2007–2014 with a focus in August 2014, when two HWs were reported. A correlation analysis of the hourly data showed medium positive correlation of ozone measurements with wind speed (r = 0.40) under NHW conditions which became stronger during the night (r = 0.49) and under HW conditions (r = 0.53 and r = 0.61 at daytime and night-time respectively). Figure 3f revealed a generally decreased wind speed under heatwave conditions for years 2007–2014 resulting to stagnant conditions that favoured ozone formation and therefore resulting to increased correlation coefficients with ozone. Focusing, on summer 2014, only 91(4%) of 2208 hourly ozone measurements were found over 120 μg/m^3^ but not for 8 consecutive hours, as suggested by the EU maximum guidelines. Figure 6 illustrates the daily variation of ozone over a period of 20 days in August, enclosing in the red areas the two reported HWs; 11-15/08/2014 and 22-26/08/2014. Almost all days of the HWs ozone concentration levels at noon exceeded the 120 μg/m^3^ threshold limit. Wind speed followed a diurnal cycle with lower values in the early morning and higher values at noon.

The diurnal profiles in Fig. 3a–f revealed lower wind speed and higher NOx concentration at 7–8 am indicating that the combination of stagnant conditions and NOx concentrations favours chemical reactions and ozone depletion, whereas at 1 pm for lower wind speed (stagnant conditions) lower NOx concentrations and higher temperatures increased ozone formation occurred. This is also observed at Fig. 6, as increased NO_2_ concentration levels (green line) occurred under HW conditions resulting to several lows in ozone levels and the diurnal slightly lower wind speed favoured stagnant conditions and ozone formation or depletion at 1 pm or 7–8 am respectively.

**Figure 7:**
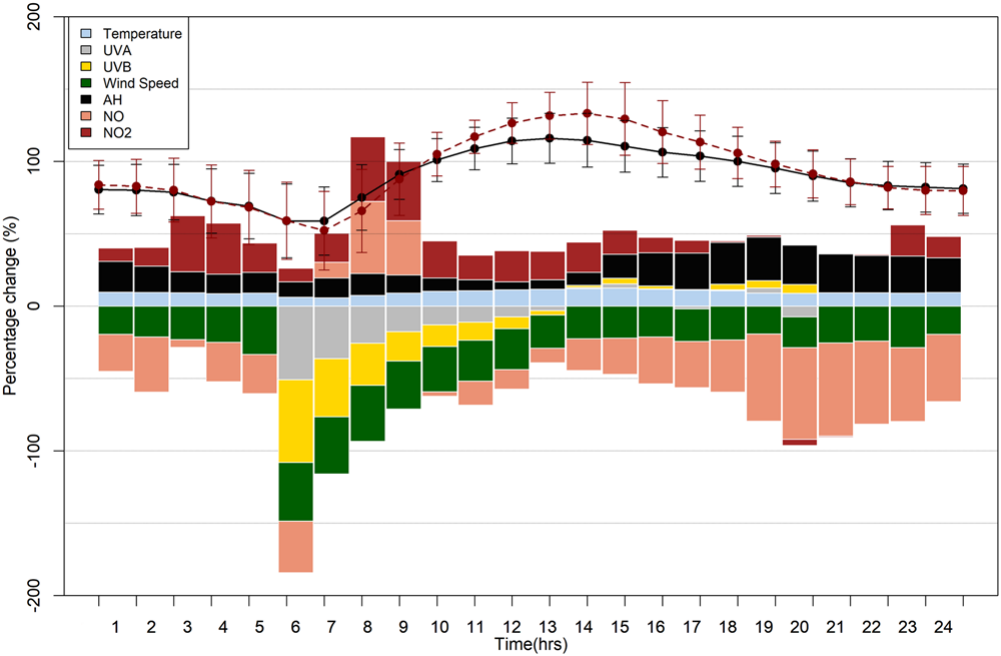
Barplot of percentage difference of the diurnal variation of parameters of Table 2 and ozone concentration levels under (**a)** NHW (black solid line) and (**b**) HW (red dotted line) conditions for years 2007–2014.

Figure 7 presents the diurnal percentage change of several parameters characterizing HW conditions and other parameters that affect chemical reactions for ozone formation or depletion, with respect to NHW conditions. Table 2 shows the numerical values of the percentage change of these parameters with respect to NHW summer conditions. Decreased ozone values at 7–8 am were accompanied by decreased UVA (~−30%), UVB (~−35%) and wind speed (~−39%) and increased absolute humidity (~15%), NO (~40%) and NO_2_ (~38%) concentration levels. At 2–3 pm increased ozone levels (~17%) were accompanied by lower NO concentration levels (~−23%) and higher UVA (~2%), UVB (~3%) and temperature (~12%). UVA and UVB percentage changes were not calculated for night-time (9 pm–6 am).

**Table 2:** Percentage change of ozone, temperature, UVA, UVB, wind speed, absolute humidity, NO and NO_2_ under HW conditions compared to NHW conditions for years 2007–2014.

Time	Ozone (μg/m^3^)	Temperature (°C)	UVA (W/m^2^)	UVB (W/m^2^)	Wind speed (m/s)	Absolute Humidity (g/m^3^)	NO (μg/m^3^)	NO_2_ (μg/m^3^)
01:00	4.12	9.56			−19.37	21.45	−25.75	9.17
02:00	3.29	9.43			−21.22	18.28	−38.11	12.88
03:00	1.83	9.14			−23.19	14.62	−5.33	38.75
04:00	−0.28	8.53			−25.01	13.64	−27.18	35.25
05:00	−1.14	8.97			−33.37	14.41	−26.99	20.28
06:00	0.23	6.07	−50.83	−57.14	−40.64	10.71	−35.56	9.45
07:00	−11.26	5.84	−36.34	−40.06	−39.65	13.73	10.79	20.07
08:00	−12.31	7.49	−25.61	−28.99	−38.85	15.01	49.77	44.86
09:00	−3.59	9.07	−17.55	−20.43	−33.18	12.55	37.44	40.88
10:00	3.92	10.29	−12.8	−15.14	−31.17	9.23	−3.22	25.57
11:00	7.41	10.65	−10.97	−12.53	−28.23	7.7	−16.73	16.89
12:00	10.73	11.23	−7.43	−8.13	−28.2	5.59	−13.52	21.42
13:00	13.46	11.79	−3.07	−2.98	−22.95	6.41	−10.22	19.82
14:00	16.22	12.15	0.82	1.49	−22.56	9	−21.79	20.74
15:00	17.19	12.58	2.78	3.92	−22.18	16.62	−24.86	16.59
16:00	13.2	11.75	0.21	2.01	−21.27	22.84	−32.33	10.69
17:00	9.29	11.17	−1.91	0.33	−22.44	25.25	−32.07	8.85
18:00	5.75	10.58	0.78	3.81	−23.33	28.88	−36.04	1.07
19:00	2.87	9.11	3.36	5.26	−19.31	30.08	−60.17	1.12
20:00	1.71	8.8	−7.4	6.25	−21.18	27.15	−63.36	−4.25
21:00	0.73	9.26			−25.49	26.79	−64.2	−0.95
22:00	−1.63	9.21			−24.06	25.89	−57.42	0.75
23:00	−2.74	9.03			−28.66	25.56	−51.08	21.68
00:00	−1.87	9.44			−19.43	23.93	−46.49	14.83

## Discussion

The results from the present analysis could improve our understanding of near-surface ozone with regards to chemical precursors, catalytic factors and physical processes; vertical and horizontal advection. Ground-level ozone concentrations showed a steady mean yearly profile for the seven investigated years (2007–2014) and similar monthly variability peaking in July, while the diurnal seasonal cycle exhibited a minimum in the morning and peaked at noon.

In the previous section the CHEM, VADV and HADV factors were analysed under HW and NHW conditions; indicating that all the observed parameters (temperature, absolute humidity, UVA, UVB and wind speed) had an effect, evident by the noteworthy correlation, on ozone measurements with increased effect under HW conditions when the synoptic situation favoured stagnant atmospheric conditions.

It is important to understand that several morphological parameters and anthropogenic activities affect the ozone concentration levels; therefore it is difficult to attribute ozone concentration levels to individual factors. The analysis of synoptic maps showed a steady state during the entire summers over the eastern Mediterranean favouring downward air movement but also indicating that near-surface ozone spikes were not attributed to VADV.

The parameters characterizing HW conditions (low wind speed, high temperatures, high water vapor) in Cyprus also assist in the formation and depletion of ozone as the stagnant conditions and the higher temperatures fasten chemical reactions in the presence of medium-high NOx concentration levels, water vapor (absolute humidity) and UV radiation. High temperature, low wind speed, UVA and UVB were considered as ozone enhancement factors due to their positive correlation with ozone, whereas higher NOx and absolute humidity were considered as ozone reduction factors. Increasing temperature is usually associated with increasing ozone because it directly influences chemical kinetic rates and the mechanism pathway for the generation of ozone but this is not always valid because the rate of increase depends also to the concentration levels of VOCs and NOx. Each variable leads to a unique, non-linear response with ozone through its particular mechanism that may affect chemical reactions for formation or depletion of ozone and may even reach a plateau level of effect such as in the research by Steiner *et al*.^29^ who noted a plateau in ozone levels above 34 °C^29^.

The photochemical reactions for ozone formation or destruction declined rapidly at night-time resulting in lower levels of ozone at night. Overall the analysis of this paper showed a noteworthy positive correlation of ozone with temperature, UVA and UVB which increased under HW conditions and was higher at daytime. Under HW conditions daily mean temperature was increased by 9.63% compared to NHW conditions, whereas the rest of the enhancement parameters remained steady or slightly decreased. Also, a decrease of near-surface ozone levels around 6:00 am and an increase from 10:00 am until 6:00 pm was noted. The high ozone measurements were positively correlated with HADV insinuating transportation of air from other areas with increased ozone concentration levels.

The higher decrease in ozone measurements in the morning (around 7:00 am) under HW conditions was attributed to the enhancement of the NOx budget (increase by about 13% of mean daily values under HW conditions). Increase of the NOx budget may have resulted to strengthening of O_3_ titration process leading to lower O_3_ measurements. However, the NOx and ozone relationship is not linear therefore simply controlling the NOx budget may be ineffective in controlling the ozone levels.

Relative and absolute humidity can affect ultraviolet radiation because of aerosol hygroscopicity^30^ and in the eastern Mediterranean atmosphere the moisture-absorbing aerosols have a greater attenuation effect on UV flux and, therefore, on ozone photochemical production but also on photochemical ozone loss (e.g. Chapter 5 in^31^). Moreover, higher absolute humidity (AH) under HW conditions suggests mixing with wetter air masses and higher availability of OH and HO_2_ radicals originating mainly from atmospheric oxidation of water vapor. Jacob and Winner^32^ noted weak sensitivity of ozone with water vapor and particularly for low pollution areas an inversely proportional relationship and for high pollution areas a proportional relationship as OH radicals react with VOCs and CO to produce ozone^21^. In the polluted atmosphere of eastern Mediterranean, Georgiou *et al*.^33^ identified high concentrations of ozone, NOx and CO in summer 2014 based on observational and simulation data, strengthening the argument of reaction of OH radicals with VOCs and CO to produce ozone.

Overall, the results highlighted that the large scale phenomenon of HWs has a profound effect on ozone concentrations, with the lowest ozone measurements occurring for higher NOx budget and absolute humidity in the morning and the highest ozone records at noon attributed to higher temperatures. This newfound conjecture encourages deeper research, combining model and observational data^33,34^, into the existent long-term weather conditions prevalent during HWs for the understanding of increase of ozone formation at daytime under higher temperatures.

## Methods

### Study area and datasets

**Figure 8:**
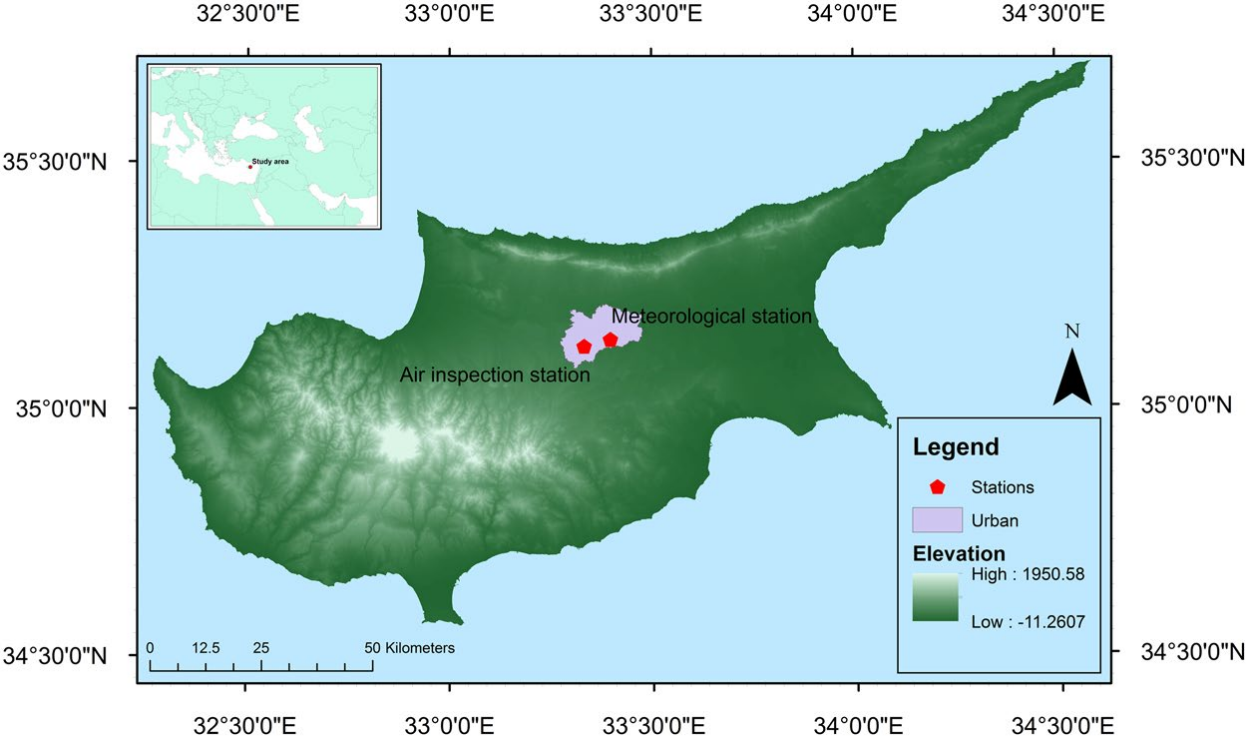
Map of Cyprus with urban area (purple area), air inspection and meteorological stations created in ArcGIS software version 10.3 (www.ESRI.com).

Cyprus (Fig. 8) is an island in the eastern basin of the Mediterranean Sea of area 9,251 km². One suburban air quality inspection station (35.1269°N, 33.3314°E) and one suburban meteorological station (35.1408°N, 33.3964°E) in the residential area of Nicosia were chosen for investigation.

Hourly air quality (ozone [μg/m^3^], NO [μg/m^3^] and NO_2_ [μg/m^3^]) and meteorological (temperature [°C], relative humidity [%], UVA [W/m^2^], UVB [W/m^2^] and wind speed [m/s]) measurements were obtained for a seven year time period, from 2007 to 2014 with about 2.5% of gaps due to missing data or outliers.

### Analysis

Using the available hourly datasets from 2007 to 2014 the next steps were followed: (1) definition of the mean yearly, monthly and diurnal concentration levels of near-surface ozone in Nicosia, (2) identification of variation in correlation coefficients between investigated meteorological and air quality parameters for summer under non heatwave(NHW) and heatwave(HW) conditions; 4 consecutive days with daily maximum temperature over 39 °C, (3) investigation of ozone under HW conditions with consideration of dominant synoptic weather conditions (horizontal advection (HADV), vertical advection (VADV), and other air quality and meteorological parameters affecting chemical reactions (CHEM) for ozone production and/or destruction.

### Data availability

The datasets generated and analysed during the current study are available from the corresponding author on reasonable request and with permission of the Republic of Cyprus Ministry of Agriculture, Rural Development and. Environment (MARDE) for the meteorological data and the Ministry of Labour, Welfare and Social Insurance for the air quality data.
